# Over-the-Counter Hearing Aids Versus Traditional Hearing Aids in Patients With Mild-to-Moderate Hearing Loss: Protocol for a Noninferiority Randomized Controlled Trial

**DOI:** 10.2196/59894

**Published:** 2024-10-25

**Authors:** Ga-Young Kim, Mini Jo, Young Sang Cho, Il Joon Moon

**Affiliations:** 1 Hearing Research Laboratory Samsung Medical Center Seoul Republic of Korea; 2 Medical Research Institute Sungkyunkwan University School of Medicine Suwon Republic of Korea; 3 Department of Digital Health SAIHST Sungkyunkwan University Suwon Republic of Korea; 4 Department of Otorhinolaryngology - Head and Neck Surgery Sungkyunkwan University School of Medicine Samsung Medical Center Seoul Republic of Korea; 5 SAIHST Sungkyunkwan University Suwon Republic of Korea

**Keywords:** hearing aids, over-the-counter hearing aids, correction of hearing impairment, randomized controlled trial, evidence-based medicine, hearing loss, aging, hearing sensitivity, mobile phone

## Abstract

**Background:**

With the aging of society, the prevalence of hearing loss (HL) is increasing. Currently, approximately 5% of the global population has HL, and this number is projected to reach 7 million by 2050. Although hearing aids (HAs) are the primary treatment for HL, their use is limited by barriers such as high costs and social stigma. To address these limitations, over-the-counter (OTC) HAs have been introduced, but their effectiveness and drawbacks require further investigation.

**Objective:**

This study aims to conduct a noninferiority randomized controlled trial comparing OTC HAs with traditional HAs to assess the clinical effectiveness of OTC HAs.

**Methods:**

We designed a noninferiority randomized controlled trial comparing OTC HAs and traditional HAs in adults with mild-to-moderate HL. A total of 64 participants (32 per group) will be recruited. Randomization will be performed using block randomization (block sizes of 2 or 4) with an equal allocation ratio. The study will include 2 types of HAs: an OTC HA (Jabra Enhance Pro) and a traditional HA (LiNX Quattro LE561-DRW) by GN ReSound A/S. OTC HAs will be self-fitted using a smartphone app, while traditional HAs will be fitted by a licensed audiologist using the National Acoustics Laboratories–Non-Linear Prescription, second generation. Assessments, including functional gain, real-ear measurement, speech audiometry, and questionnaires, will be conducted at 6-month intervals over the course of 3 visits. Statistical analysis will compare the 2 outcomes, focusing on functional gain, to determine noninferiority.

**Results:**

This study is scheduled to begin in August 2024 and has not yet recruited any participants. The study will be conducted over 2 years, from August 2024 to July 2026. Each participant will have 2 follow-up visits at 6-month intervals, making the total follow-up period 1 year.

**Conclusions:**

Since 2022, the introduction of OTC HAs has revolutionized access to these devices. Researchers, clinicians, and the general public are keen to evaluate the clinical effectiveness of OTC HAs, as more individuals will likely use them for HL. This increased usage will provide valuable real-world data to understand the benefits and limitations of OTC HAs. Monitoring the outcomes and user feedback will provide insights into their effectiveness and impact on hearing rehabilitation.

**International Registered Report Identifier (IRRID):**

PRR1-10.2196/59894

## Introduction

### Background

As society undergoes a rapid transition toward an aging population, the prevalence of hearing loss (HL) is significantly increasing. According to the World Health Organization, approximately 5% of the global population (4.3 million individuals) currently has HL, and this number is projected to reach 7 million by 2050 [1]. One of the reasons HL has attracted attention recently is due to its association as the most modifiable risk factor for dementia [2]. Therefore, proactive hearing rehabilitation is crucial.

Hearing aids (HAs) serve as the first-line management for patients with HL. However, several barriers can prevent people from using HAs. These barriers include economic limitations such as high costs (ranging from US $3000 to US $6000 a pair), limited insurance coverage, and social stigma [3-6]. As evidence of these barriers, one study demonstrated that the rate of purchasing HAs among individuals with HL was 17.4%, whereas the use rate was merely 12.6% [7].

In an effort to increase accessibility to HAs, the US Food and Drug Administration (FDA) has recently created a regulatory category for over-the-counter (OTC) HAs. These are classified as a type of medical device that can be purchased without requiring a prescription or consultation with licensed hearing health care professionals (HHPs) [8]. Unlike traditional HAs, OTC HAs are specifically designed for adult patients with mild-to-moderate HL. The introduction of OTC HAs is anticipated to broaden access to affordable hearing care for millions of patients who currently have HL but are not adequately served by the traditional HA market.

The market for OTC HAs has been expanding rapidly. However, due to the recent FDA approval, empirical research is not available regarding their clinical effectiveness. Furthermore, OTC HAs come with multiple disadvantages that require thorough validation. One prominent drawback is that patients are responsible for fitting the HAs themselves, potentially leading to reduced accuracy in amplification [9]. In addition, the predominant older adult population, who may lack technical proficiency, may encounter difficulties in operating OTC HAs. Consequently, there is a need for studies aimed at demonstrating that OTC HAs are not inferior to traditional HAs.

To address this, we aim to conduct a noninferiority randomized controlled trial (RCT) comparing OTC HAs with traditional HAs. We hypothesize that OTC HAs will demonstrate noninferiority when compared with traditional HAs.

### Objectives

The primary objective of this study is to compare the effects of OTC HAs and traditional HAs on patients with HL. The primary outcome of interest is functional gain at the conclusion of a 1-year intervention period. Secondary objectives include assessing the impact of OTC HAs and traditional HAs on speech audiometry in both quiet and noisy environments, conducting real-ear measurement (REM), and administering questionnaires to gather additional information.

## Methods

### Study Design and Setting

**Figure 1:**
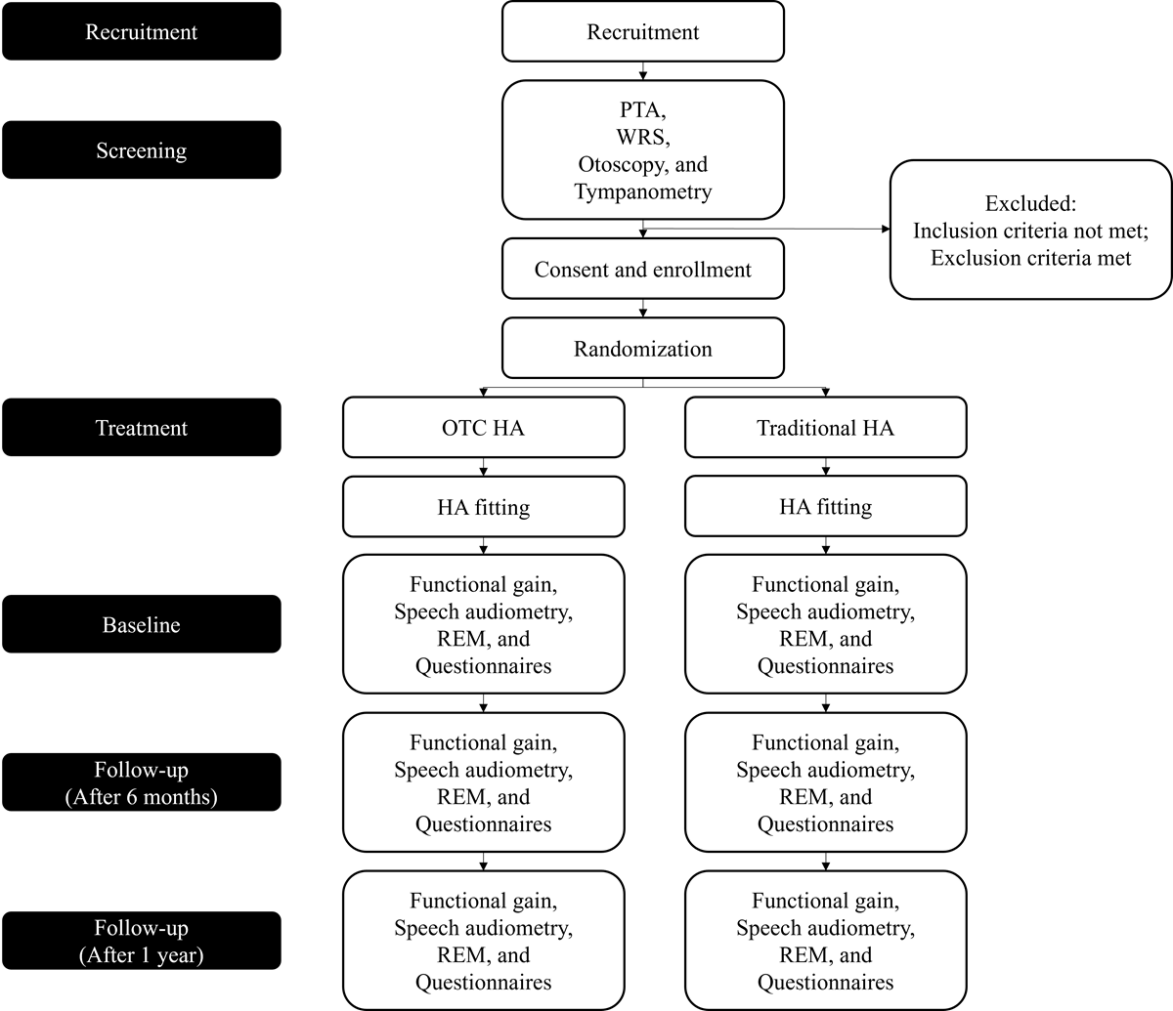
Consolidated Standards of Reporting Trials (CONSORT) diagram. HA: hearing aid; OTC: over-the-counter; PTA: pure-tone audiometry; REM: real-ear measurement; WRS: word recognition score.

In this noninferiority RCT, patients will be randomly assigned to 2 groups. The study will take place at a tertiary hospital in South Korea, serving as a single center for the research. The protocol for this study follows the guidelines outlined in the Standard Protocol Items Recommendations for Interventional Trials statement [10] (Figure 1).

### Study Population

To be eligible for this study, participants must meet specific inclusion and exclusion criteria (Textbox 1).

**Inclusion and exclusion criteria.**

**Inclusion criteria**

- Voluntary willingness and ability to participate
- Aged 18 years or older
- Proficiency in the Korean language
- Hearing threshold ranging from 26 dB to 55 dB (indicating mild-to-moderate hearing loss) based on the average of 4 frequencies (0.5, 1, 2, and 4 kHz) in both ears
- Absence of significant abnormalities in the outer ear and middle ear as determined by otoscopy
- Type A tympanogram
- No previous history of sudden hearing loss

**Exclusion criteria**

- Mental incapacity to participate in this study
- Previous use of hearing aids
- Absence of outer or middle ear diseases

### Sample Size

The number of participants for this study was determined using G*Power (version 3.1.9.4) software (Institute for Experimental Psychology). The estimated sample size was based on the findings of a previous study [11]. The calculated total sample size was 56 participants, considering an effect size (Cohen *d*) of 0.9, an error probability of .05, and a power (1– error probability) of .95. Therefore, considering an attrition rate of 15%, a total of 64 participants will be recruited, with 32 participants allocated to each group.

### Recruitment

Announcements regarding the study will be disseminated through hearing-related support groups, audiology organizations, newsletters (daily newspapers, magazines, etc), hearing clinics of primary and secondary hospitals, otorhinolaryngology outpatient clinics of a tertiary hospital, and social media platforms (Facebook [Meta], Instagram [Meta], X [formerly known as Twitter], etc). Individuals who express interest will receive information and undergo a screening test. If they meet the inclusion criteria, they will be enrolled in this study.

### Assignment to Intervention Groups

After inclusion, the patients will be randomly assigned to 1 of 2 intervention groups: OTC HAs or traditional HAs. Randomization will be performed electronically using R software (R Core Team) with block randomization and an equal allocation ratio of 1:1. The block sizes will be 2 and 4. The researchers will remain blinded to the randomization sequence. However, due to the nature of the intervention, it is not possible to blind the participants and care providers to their assigned group.

### Data Collection Methods

The results will be recorded on paper and then transferred to a Microsoft Excel document for data entry. These files will be stored as electronic records for archiving purposes.

### Data Management

The data will be securely stored on a designated server and access will be strictly controlled and limited to authorized personnel authorized by the institutional review board (IRB). Only individuals with explicit permission will have the authority to manage and access the recorded data.

### Statistical Analysis

In the descriptive analysis, continuous data will be presented using means or medians, depending on their normality. Categorical data will be quantitatively presented, including the number and percentage for each variable. For primary and secondary outcome variables, if the normality assumption is met, repeated measurements of analysis of variance will be conducted. If the normality assumption is not met, the Friedman test will be used. Independent variables will be types of HAs and time, while the dependent variables will be outcome variables. Post hoc analysis using Bonferroni correction will be conducted for significant variables. If there is no significant difference in the primary outcome of functional gain between the 2 HAs, it will be considered noninferior.

### Interventions

The study involves 2 types of HAs: (1) an OTC HA (Jabra Enhance Pro; GN ReSound A/S) and (2) a traditional HA (LiNX Quattro LE561-DRW; GN ReSound A/S). Both HAs used in the study are receiver-in-canal type devices and are manufactured by the same company. The OTC HAs will be fitted by the patients themselves following the manufacturer’s guidelines through a dedicated smartphone app. On the other hand, the traditional HAs will be fitted by a licensed audiologist using the National Acoustics Laboratories–Non-Linear prescription, second generation (NAL-NL2). All HA settings, such as noise reduction, directionality, and feedback cancellation, will be set to their default values.

### Outcome Measurements

The participants will be screened using otoscopy, tympanometry, PTA, and WRS at the first visit (baseline). The participants who pass the screening tests will undergo main tests, including functional gain, REM, speech audiometry, and questionnaires at every visit.

#### Screening Tests

##### PTA Assessment

PTA will be conducted to determine participants’ hearing sensitivity. PTA will be conducted using an audiometer (GSI Audio Star audiometer; Grason-Stadler Inc) with TDH-39 headphones in a double-walled soundproof booth. PTA measurements will be performed in each ear at 0.25 kHz and 8 kHz frequencies following standard procedures [12]. The participants will respond by pressing a button each time they hear the tone. A total of 4 frequency averages (0.5, 1, 2, and 4 kHz) will be calculated.

##### WRS Assessment

WRS provides insight into the participants’ ability to discriminate speech sounds. In this study, WRS will be measured using the same audiometer used for PTA with the TDH-39 headphone in a double-walled soundproof booth. A compact disc player (Yamaha TSX-B232; Yamaha Corporation) will be connected to the audiometer. A total of 25 monosyllabic words [13] at the most comfortable level will be presented. The participants will be asked to repeat the word back to the tester. Percentage-correct scores will be calculated for scoring.

##### Otoscopy

Otoscopy (mini 3000 F.O.; HEINE Optotechnik GMbH and Co) will be used to visually examine structures of the external ear canal, tympanic membrane, and middle ear. The audiologists will insert the cone of the otoscope into the participants’ ear canal.

##### Tympanometry

Tympanometry is an objective, physiological measurement of acoustic admittance of the middle ear as a function of air pressure in a sealed ear canal. A tympanometry will be conducted with a tympanometer (Titan; Interacoustics A/S). The audiologist will insert the probe tip into the participants’ ear canal to measure the air pressure produced by the tympanometer. The tympanogram will illustrate positive and negative variations in air pressure.

#### Main Tests

##### Functional gain

Functional gain will be measured by comparing the hearing thresholds at predetermined frequencies before and after wearing the HAs in a double-walled soundproof booth. The functional gain is theoretically equivalent to the real-ear insertion gain measured through REM. By comparing the functional gain at each frequency with the fitting formula for HA, we can determine the appropriateness of the HA fitting.

##### Speech Audiometry

In quiet conditions, a different list from the Korean standard monosyllabic word lists for adults [13] used in WRS will be used. The testing will be conducted in a free field sound, where 25 target monosyllabic words will be presented at a level of 50 dB HL. The participants will be instructed to repeat the words. The presentation of the target sounds will be positioned 1 meter away from the participant, facing them directly (0°). The percentage of correct responses will be compared before and after wearing HAs. In noisy conditions, the Korean version of the Hearing In Noise Test (K-HINT) will be used [14]. The K-HINT presents speech spectrum noise from front and lateral speakers. For each condition, the noise level is set to 65 dBA, while the speech level is adjusted to measure the reception threshold for speech (RTS). The RTS is determined by measuring the signal-to-noise ratio at which target sentences are perceived correctly 50% of the time. RTS will be compared before and after wearing HAs.

##### REM Assessment

We will use the Aurical Free Fit device (Natus) for REM. After fitting the HAs on the patient, a probe tube will be inserted near the tympanic membrane within a proximity of 6 mm. The REM process involves entering the PTA results into the REM software, selecting the fitting formula (NAL-NL2), and comparing the target gain derived from the fitting formula with the real-ear insertion gain obtained from the REM. During the measurements, participants will hear the stimulus sound from a speaker placed 1 meter away at 0° azimuth. The stimulus sound used will be an Inter Speech Test Signal presented at an intensity of 65 dB SPL, which is comparable with the average level of conversational speech.

##### Questionnaires

The Korean version of the Abbreviated Profile of Hearing Aid Benefit (K-APHAB) consists of a total of 24 items divided into 4 subscales of ease of communication, reverberation, background noise, and aversiveness to measure HL in everyday situations. For each item, a 7-point Likert scale is used (always=1 [99%]; never=7 [1%]). The benefit of the HA will be calculated as unaided scores minus aided scores [15]. The Korean version of the international outcome inventory for hearing aids (K-IOI-HA) is composed of 7 questions designed to subjectively evaluate the performance of HAs solely based on daily use, benefit, residual activity, satisfaction, residual participation restrictions, impact on others, and quality of life. Each item is rated on a 5-point scale, with higher scores indicating greater benefit. The total score ranges from 0 to 35 points [16].

### Participant Timeline

All participants will undergo in-person assessments over the course of 3 visits conducted at 6-month intervals. During the first visit, the participants will be screened and randomly assigned to 1 of 2 groups: OTC HAs and traditional HAs. Each HA will be fitted according to the guidelines described in the “Interventions” section before conducting the main tests. Subsequently, the participants will undergo main tests and be instructed to use the HA for at least 6 hours daily throughout the intervention period. After 6 months, the participants will return to the clinic for assessments using the same tests from the first visit as an intermediate evaluation. During the final visit (after 6 months), the participants will undergo the same evaluations as during the second visit and return the HAs. If participants experience any discomfort or issues with HA usage during the study period, they will be encouraged to visit the clinic at any time for adjustments (Table 1).

**Table 1:** Timeline for this study.

Outcomes					Screening and baseline			After 6 months			After 1 year			
**Study consent**					✓			—^a^			—			
**Screening tests**														
	PTA^b^		✓			—			—					
	WRS^c^		✓			—			—					
	Otoscopy		✓			—			—					
	Tympanometry		✓			—			—					
**HA^d^ provision and fitting**				✓			✓			—				
**Main tests**														
	**Functional gain**		✓			✓			✓					
	**Speech audiometry**													
		Quiet	✓			✓			✓					
		Noisy (K-HINT^e^)	✓			✓			✓					
	**REM^f^**		✓			✓			✓					
	**Questionnaires**													
		K-APHAB^g^	✓			✓			✓					
		K-IOI-HA^h^	✓			✓			✓					
**HA return**					—			—			✓			

^a^Not applicable.

^b^PTA: pure-tone audiometry.

^c^WRS: word recognition score.

^d^HA: hearing aid.

^e^K-HINT: Korean version of the Hearing In Noise Test.

^f^REM: real-ear measurement.

^g^K-APHAB: Korean version of the Abbreviated Profile of Hearing Aid Benefit.

^h^K-IOI-HA: Korean version of the International Outcome Inventory for Hearing Aids.

### Ethical Considerations

The study will be conducted in accordance with the principles outlined in the Declaration of Helsinki. Furthermore, it has received approval from the Institutional Review Board (IRB) of Samsung Medical Center (IRB 2023-04-149). Informed consent must be obtained prior to any clinical trial-related activities. Before participants join the clinical trial, the researcher will explain the trial process to them, obtain a signed consent form, and provide a copy of the consent form to the participants. Participation in the trial is voluntary, and consent can be withdrawn at any time.

To ensure privacy and confidentiality, no personally identifiable information will be recorded in any documents where trial results are documented, and participant identification will be coded. Screening numbers will be assigned sequentially as OTC_01, _02, etc, and documents linking the code to participant identities will be attached to the researcher’s file and stored in a locked cabinet. All clinical trial data collected and stored at the research institution may be kept for up to three years after the trial concludes.

If participants agree to take part in the study, they will not incur any costs for the tests conducted as part of the trial; however, they will be responsible for hospital admission fees, any tests unrelated to the trial, and consultation fees. The trial involves three visits in total, and participants will receive $50 for transportation costs at each visit. However, if a participant does not meet the inclusion criteria, drops out, or withdraws from the trial, transportation costs will not be provided.

## Results

This study is scheduled to begin in August 2024. The trial is currently in the recruitment phase after receiving IRB approval; however, no patients have been enrolled yet. The study will be conducted over 2 years, from August 2024 to July 2026. Each participant will have 2 follow-up visits at 6-month intervals, making the total follow-up period 1 year.

## Discussion

### Expected Findings

The purpose of this study is to conduct a noninferiority RCT comparing OTC HAs with traditional HAs to assess the clinical effectiveness of OTC HAs. The expected findings of the study are that OTC HAs will demonstrate noninferiority to traditional HAs in terms of functional gain. This means that OTC HAs will provide similar performance to traditional HAs. Recent research has focused on the clinical effectiveness of personal sound amplification products; however, there is still a lack of studies on the clinical effectiveness of OTC HAs, as they have only recently been introduced. To the best of our knowledge, this study is the first to investigate and potentially confirm the noninferiority of OTC HAs compared with traditional HAs. These findings could have significant implications for the accessibility and affordability of HAs, providing more options for individuals with HL.

### Strength and Limitations

The strengths of this study include its innovative approach, being the first to conduct a noninferiority RCT comparing OTC HAs with traditional HAs, thus addressing a significant gap in the existing literature. The clinical relevance is noteworthy, as assessing the clinical effectiveness of OTC HAs provides valuable insights that could influence clinical practice and policymaking. Furthermore, the findings from this study could have substantial implications for the accessibility and affordability of HAs, potentially offering more affordable options for individuals with HL. The study uses rigorous RCT methodology to ensure reliable and valid results, enhancing the credibility of the findings. Finally, the study’s timeliness is significant, as OTC HAs have only recently been introduced, and this research addresses a current need for evidence on their clinical effectiveness.

The limitations of this study include a short-term focus, as it may primarily address short-term outcomes, potentially overlooking longer-term effectiveness and user satisfaction with OTC HAs. In addition, the generalizability of results may be limited to specific populations and settings and may not extend to all individuals with HL or different health care systems. There is also a risk of potential bias, as with any RCT, where selection bias or other unforeseen biases could affect the results. Furthermore, technological variability in the OTC HAs available on the market could influence the outcomes, making it challenging to generalize findings across all OTC HAs. Finally, the study’s scope may be limited, as it may not address all potential factors influencing the effectiveness of HAs, such as user preferences, ease of use, or long-term adherence to using the devices.

### Conclusions

Since 2022, the introduction of OTC HAs has brought significant developments in the field. The availability of OTC HAs has resulted in a notable shift in how patients with HL can access and obtain these devices. With OTC HAs now being sold directly to consumers without the need for a prescription from HHPs, there is growing anticipation and curiosity surrounding their clinical effectiveness. Researchers, clinicians, and the general public are keen to explore the extent to which these devices can provide meaningful improvements in hearing ability and overall quality of life. As a result, there is a previous demand for studies to evaluate the clinical effectiveness of OTC HAs. With the wider availability and accessibility of OTC HAs, it is anticipated that more individuals will be inclined to try these devices as a potential solution for their HL. The increased usage will likely contribute to a wealth of real-world data and experiences that can be analyzed to further understand the benefits and limitations of OTC HAs. Researchers and HHPs will closely monitor the outcomes and feedback from users to gain insights into the effectiveness, user satisfaction, and overall impact of OTC HAs on hearing rehabilitation.

## Abbreviations

HA: hearing aid
HHP: hearing health care professional
HL: hearing loss
IRB: Institutional Review Board
K-APHAB: Korean version of the Abbreviated Profile of Hearing Aid Benefit
K-HINT: Korean version of the Hearing In Noise Test
K-IOI-HA: Korean version of the International Outcome Inventory for Hearing Aids
NAL-NL2: National Acoustics Laboratories–Non-Linear Prescription, second generation
OTC: over-the-counter
PTA: pure-tone audiometry
RCT: randomized controlled trial
REM: real-ear measurement
RTS: reception threshold for speech
WRS: word recognition score

## Data Availability

Data sharing is not applicable to this article as no datasets were generated or analyzed during this study.
